# A comprehensive review of urologic complications in patients with diabetes

**DOI:** 10.1186/2193-1801-3-549

**Published:** 2014-09-23

**Authors:** Fernando Arrellano-Valdez, Marta Urrutia-Osorio, Carlos Arroyo, Elena Soto-Vega

**Affiliations:** Facultad de Medicina, Universidad Popular Autónoma del Estado de Puebla, 21 sur 1103, Barrio de Santiago, Puebla, Puebla C.P 72410 México; Hospital Ángeles de Puebla, Puebla, México; Hospital Universitario de Puebla, Benemérita Universidad Autónoma de Puebla, Avenida 25 Poniente 1301, Los Volcanes, 72410 Heroica Puebla De Zaragoza, PUE Mexico

**Keywords:** Diabetes mellitus, Urologic diseases

## Abstract

Diabetes Mellitus (DM) is a chronic disease characterized by hyperglycemia, as a result of abnormal insulin production, insulin function, or both. DM is associated with systemic complications, such as infections, neuropathy and angiopathy, which involve the genitourinary tract. The three most significant urologic complications include: bladder cystopathy, sexual dysfunction and urinary tract infections.

Almost half of the patients with DM have bladder dysfunction or cystopathy, which can be manifested in women as hypersensitivity (in 39–61% of the diabetic women) or neurogenic bladder. In males it can be experienced as lower urinary tract symptoms (in 25% of diabetic males with a nearly twofold increased risk when seen by age groups). Additionally, an increased prostate volume affects their micturition as well as their urinary tract.

Involving sexual dysfunction in women, it includes reduced libido, decreased arousal, clitoral erectile dysfunction and painful or non-sensitive intercourse; and in diabetic males it varies from low libido, ejaculatory abnormalities and erectile dysfunction. Globally, sexual disorders have a prevalence of 18–42%. Erectile dysfunction is ranked as the third most important complication of DM. Urinary tract infections are observed frequently in diabetic patients, and vary from emphysematous infections, Fournier gangrene, staghorn infected lithiasis to repetitive bacterial cystitis. The most frequent finding in diabetic women has been lower urinary tract infections. Because of the high incidence of obesity worldwide and its association with diabetes, it is very important to keep in mind the urologic complication associated with DM in patients, in order to better diagnose and treat this population.

## Introduction

The high incidence of urologic complications associated with diabetes mellitus (DM) is an increasing health concern throughout the entire world. Especially, if we consider the high incidence of obesity worldwide, being the United States and Mexico the countries with major incidence (Rull et al. 2005).

DM is a group of metabolic diseases characterized by hyperglycemia caused by a diminished insulin secretion, insulin effect, or both. This chronic hyperglycemia is associated with systemic long-term damage, dysfunction, and failure of several tissues. DM is classified into many categories, being the most frequent: type 1, type 2 and gestational diabetes (American Diabetes Association 2011).

Urologic complications in patients involve the endothelial and neural damage associated systemically, which involve the genitourinary tract. To simplify this review we divided them into three groups: lower urinary tract dysfunction, sexual dysfunction, and urinary tract infections. It is noteworthy to mention that all of them have a significant negative effect on the quality of life of diabetic patients, as well as life threatening complications.

## Urologic complications in patients with diabetes

### Bladder dysfunction

Almost half of male and female patients with DM suffer from different degrees of bladder cystopathy. Bladder dysfunction (BD) can involve different degrees and combinations of diminished bladder filling sensation and poor contractility, which results in an increased post void residual urine, predisposing to infections, lithiasis or renal damage.

A number of clinical studies in women and men with diabetes have reported bladder hypersensitivity or bladder instability as the most frequent finding, ranging from 39–61% of the patients (Golbidi and Laher 2010). BD was present in 74.07% of men (DC, 50%; bladder outlet obstruction OO, 25%; detrusor overactivity, 25%) and in 59.26% of diabetic women (Kebapci et al. 2007). Additionally, recent evidence suggests that lower urinary tract symptoms (LUTS) may occur more frequently among men with diabetes, with an estimated 25% to nearly twofold increased risk of LUTS in men with diabetes. There are a wide variety of symptoms which vary from urge incontinence (risk of 40% to 80%) to the most severe expression of overflow incontinence (80% increased risk) (Brown et al. 2003). Therefore, it is obvious that this pathology represents a combination of both, storage and voiding abnormalities.

Bladder symptoms are divided in irritative and obstructive. The first ones can be presented as an overactive bladder syndrome, which involves an overexcited detrusor muscle, which cause urgency, pollakiuria, nocturia and urgency incontinence. Obstructive symptoms are related to a pseudo-obstructive bladder, which represent a late phase of the diabetic neuropathy and are characterized by low uroflowmetry, high postmictional residue, and a hypotonic bladder in the cystometry. The last one reflects a myogenic alteration of the neuronal and microvasculature. These pseudo-obstructive symptoms include a decreased size and strength of the voiding flow, terminal dribbling, decreased sensation of a full bladder and high post void residual urine (very similar to those seen in the benign prostate hyperplasia) (Daneshgari et al. 2009; Liu and Daneshgari 2006).

During the early stage of diabetic cystopathy, there is an increase in bladder storage capacity, which affects its compliance or ability to adapt its fullness. Thus, diminishing its contractility, and increasing the post void residual volume. It has also been noticed that in an effort to counteract against these changes, there is certain bladder hypertrophy which causes further bladder instability in its contractions because of collagen deposits, making the tightening of the detrusor muscle ineffective. All of these deviations in the bladder physiology and function have been reproduced with cystometry in diabetic rats, which shows a bladder with increased capacity and raised threshold for initiation of micturition reflex. When sacrificed diabetic rats have bladder hypertrophy resulting in approximately a doubling of bladder weight as an effect of abnormally prolonged diuresis (Liu and Daneshgari 2006). With this in mind, it has been proposed that the associated increase in diuresis due to DM with the neural and endothelial damage, can collectively lead to the detrusor muscle hypertrophy in an attempt to adapt it. The bladder hypertrophy could also cause an increase in the oxidative stress, associated with further damage to the muscle (Satriano 2007), or it could be induced by an axonal transport deficiency in the NGF (neural growth factor) which plays an important role in inducing diabetic neuropathy, in diabetic rats (decreased NGF levels in the bladder are associated with bladder dysfunction). Bladder tissue remodeling is also associated with down regulation of TGF (tissue growth factor) and collagen mRNA levels, which produce an increase in elastin synthesis. Bladder compliance in DM patients is increased, as a result not only from a reduction in collagen synthesis but also from an increased in elastin synthesis (Gray et al. 2008).

The micturition reflex is the neural stimulus controlled by M2 and M3 receptors. In DM has been found an increased number of muscarinic receptors in the urothelium, increasing the sensory nerve activity and affecting the detrusor contraction, causing further bladder dysfunction (Cheng et al. 2007). Also the abnormalities in the Ca^++^ and K^+^ channels that further increase the overactive detrusor muscle. When cells are constantly exposed to hyperglycemia for long periods of time, it can cause an accumulation of oxidative stress products; which plays an important role in nerve damage, that cause cystopathy and erectile dysfunction (Beshay and Carrier 2004). Cystopathy has been demonstrated in a rabbit model with alloxan induced diabetes, in which Changolkar et al. observed an aldose reductase over expression and an increment in lipid peroxidation products, resulting in diminished detrusor contractility. Furthermore, this overexpression promotes the production of sorbitol, increasing glucose through the polyol pathway, these changes lead to myopathy which causes incomplete voiding during micturition. (Changolkar et al. 2005).

In women with DM the insulin treatment increases the risk of urge incontinence, compared with the use of metformin which has shown that it does not have any effect in the incontinence (Brown et al. 2004, Jackson et al. 2004).

Concerning the different types of incontinence, they are managed according to their etiology. In the asymptomatic diabetic patient, it is advisable to instruct them on schedule voiding and the association of external compression of the pelvic area to optimize their bladder emptying.

The first line of treatment of urge incontinence (result of a hyperactive detrusor) is an oral muscarinic selective anticholinergic such as oxibutynin, tolterodine or solifenacin. In certain individuals, infiltration of the detrusor muscle with botulinum toxin has proven to diminish their urge incontinence. If severe urge incontinence is not resolved with muscarinic selective anticholinergics, then a surgical approach could be offered, which includes: bladder denervation, myomectomy (to cause a pseudodiverticulum) and bladder augmentation with ileal cystoplasty. However, all of them have the risk of causing an increased post-void volume with its association with urinary tract infections. In certain males with added bladder outlet obstruction due to prostate enlargement, transurethral surgery could be considered. Moreover, there is a new drug 2-(2-aminothiazol-4-yl)-N-[4-(2-{[(2R)-2-hydroxy-2-phenylethyl] amino}ethyl) named mirabegron a β3- adrenergic agonist. It has been shown that simulating this receptor contributes to increase the urine storage, through direct relaxation of the detrusors smooth muscle (Aizawa et al. 2012); with a rapid onset of action, providing a rapid relief in the symptoms of the patient and has an excellent tolerability profile, thus helping patients persist with the treatment (Chapple et al. 2014). Although there is little real evidence about its effectiveness and side effects.

Finally, in patients with a neurogenic bladder with overflow incontinence, the first line of treatment is a cholinergic treatment with bethanechol to increase the bladder contractility and better emptying. In case of empty failure, frequent clean intermittent catheterization is the best choice to avoid long term indwelling catheter because of the risk of increased infection rate, lower urinary tract lithiasis and epidermoid bladder carcinoma (Deli et al. 2013).

### Prostatic enlargement

DM is frequently associated with benign prostatic hyperplasia (BPH), because of the age of incidence. Additionally, bladder dysfunction, could cause an increased sympathetic nerve activity and the vascular damage, resulting in further hypoxia in the bladder and prostate, associated with an abnormal cell proliferation, which added results in an increase in the lower urinary tract symptoms (Berger et al. 2005).

One of the potential explanations of the presence of BPH in diabetic patients, involves the insulin-like growth factor (IGF). In type-2 diabetes, β-cells secrete greater concentrations of insulin, the resulting hyperinsulinemia which stimulates IGF production; in mouse models of hypoinsulinemia had a significant prostate growth (Ikeda et al., 2000; Vikram et al., 2010). The type 1 diabetes models do not show an effect on prostate growth (Yono et al., 2005; Yono et al., 2008). The possible mechanism is the high degree of homology between insulin receptor and IGF receptor (Ullrich et al., 1986), it has been described that exist a cross-activity of their receptors (Frasca et al., 2008; Liu 2007). So the insulin may cause the prostatic growth during type-2 diabetes by activating the IGF receptor.

The treatment for patients with BPH depends of the prostate volume. Patients with a small prostate, alpha-1 blockers are the first line of treatment (non selective: doxazosin and terazosin; and uroselective: tamsulosin, alfuzosin and silodosin). In patients with BHP and erectile dysfunction the use of daily phosphodiesterase type 5 inhibitors have been shown to reduce both symptoms. Individuals with an enlarged prostate (over 40gr) the use of combined alpha-1 blockers with an alpha 5 reductase inhibitor (finasteride (selective) and dutasteride (non-selective) making it more effective) is recommended, in order to diminish the prostate volume in the long term with the faster effect on bladder neck relaxation. Another newly recognized group of patients are the ones with only detrusor hyperactivity, which should be treated with muscarinic selective anticholinergic drugs as mentioned above in the urge incontinence section. And finally, in case that all of the above have failed or there is an indication for surgery, the surgical approach should be done, being the gold standard the transurethral resection of the prostate (with bi-polar, mono-polar or laser energy) (Mcvary et al. 2011).

### Urethral tightening

Endothelial dysfunction and nitric oxide (NO) deficiency are among the most important factors in the development of diabetic complications, and the lower urinary tract is also affected. NO is partially responsible of the outflow obstruction by affecting the relaxation of the urethral sphincter, causing afferent neurons to be hyper-excitable in the progression of diabetes (Torimoto et al. 2004). Recent evidence has shown that the increased risk of an overactive bladder in diabetic patients is closely related to the peripheral nerve irritation, and if this occurs in males and females, then this should be taken into account when analyzing bladder dysfunction and prostate enlargement (Wei-Chia 2009). All of the above explains why diabetic patients can be successfully treated for BPH symptoms and erectile dysfunction with daily oral phosphodiesterase 5 inhibitors (currently the only available drug is 5 mg Tadalafil).

### Sexual dysfunction

Sexual dysfunctions are defined as the inability to achieve or maintain an adequate sexual response to complete a sexual encounter or intercourse resulting in a satisfactory orgasmic sensation; it affects diabetic males and females patients, and includes diminished libido, orgasmic abnormalities and erectile dysfunction. The incidence of sexual dysfunction in healthy males is up to 32% compared to 46% in type II diabetic males (Vickers and Wright 2004). In females it is harder to diagnose because of the multiple confusing factors that have an effect. However, it has been proposed that in healthy women, 25-63% have some sort of sexual dysfunction (Kammerer-Doak 2012), and in women with type 1 DM they have a prevalence of sexual dysfunction of 71% and of 42% in women with type 2 DM (Enzlin et al. 2002; Owiredu et al. 2011). These abnormalities involve a group of alterations that affect significantly their quality of life; which includes: reduced desire, decreased arousal, orgasmic abnormalities and painful intercourse. In diabetic women, clitoral erection is also affected, however it is one of the multiple components of sexual function; this is why its treatment with oral inhibitors of phosphodiesterase- 5 inhibitors does not completely solve sexual dysfunction in women. In DM it is one of the most frequent chronic complications that needs further research because its incidence, pathophysiology and treatment are partially understood and unfortunately in clinical practice very rarely questioned and treated (Doruk et al. 2005).

It is important to consider certain risk factors that further affect diabetic men and women such as: age, neurogenic and vascular complications, medications (Table 1), obstetric history, as well as their association with: cancer, cardiovascular disease, neurological conditions and hysterectomy, among others.

**Table 1:** **Medications that increase sexual dysfunction in the diabetic patient**

Diseases	Medications
Cardiovascular disease	Antihypertensive, B blockers
Chronic pulmonary disease	Methyldopa, clonidine, reserpine
Chronic renal insufficiency	Thiazide diuretics, spirinolactones
Prostatic enlargement	Phenothiazines, butyrophenones, tricyclic antidepressants, selective serotonin reuptake inhibitors
Prostate cancer	Estrogens, anti-androgens, LH antagonist.
Hypogonadism	Cimetidine, metoclopramide
Thyroid desease	Alcohol
Cancer of any origin	Radiotherapy, chemotherapy

### Erectile dysfunction

Erectile dysfunction (ED) is described as a long term persistent inability to achieve or maintain an adequate rigid erection in order to have a satisfactory sexual encounter. ED is the third most frequent complication of diabetes and is considered as one of the most important complications that affects their quality of life (Latini et al. 2003). ED manifests after 10 to 12 years since the onset of DM and commonly occurs earlier in males with DM (10 to 15 years) because of diabetic endothelial and neural damage associated with persistent high serum glucose levels, in fact Sun et al. propose that if males younger than 45 have ED it could be an early sign of DM (Sun et al. 2007).

It has been proven in experimental animal models that central and peripheral neuropathy, endothelial dysfunction and impaired neurotransmission are the underlying factors in the pathogenesis of diabetic ED.

Men with erectile dysfunction have a diminished response causing less relaxation of the vascular smooth muscle tissue, due to the deficient production of NO in the non adrenergic- non cholinergic neurons and in the endothelium (Morano 2003). There is also a significant accumulation of advanced glycation products (Seftel et al. 1997) with an altered expression of arginase, which is a competitor with the nitric oxide synthase for its substrate L-arginine (Bivalacqua et al. 2001). All of these factors cause a tendency towards vasoconstrictors, such as phenylephrine and endotheline-1, causing the lack of vasodilatation that is the cause of penile erection.

There are several mechanisms that play an important role in the pathophysiology of diabetic ED, which include: an increased flux in the hexosamine and polyol pathway, activating the protein kinase and the intracellular accumulation of advanced glycation products. The polyol pathway, forms sorbitol, by action of the aldose reductase; this sorbitol accumulates inside the cell, causing a diminished myo-inositol concentration, which is a precursor of the phosphatidylinositol, a molecule needed for the membrane Na-K ATPase bomb to function. The sorbitol increase causes a constant damage to the peripheral nerves. The Advanced glycation products AGE’s are produced by the glycation of proteins, the union of the AGE to their macrophages receptors can lead to cytokine release (Brownlee 2001, Neves 2013). It has been proposed that AGE’s produces free radicals, reactive oxygen species that produce oxidative cell damage, cGMP decrease, NO extinction and decrease in the cavernosal smooth muscle relaxation (Cartledge et al. 2001). McVary and cols argue that not only does peripheral neuropathy have a major role, but also the spinal sexual reflexes. In the study of diabetes induced model in rats, they showed a diminished copulatory behavior and penile reflexes after 4 to 12 months (Mcvary et al. 1997).

Concerning the vascular component, the endothelial damage also participates in a direct form, because the arterial inflow is diminished in diabetic patients in comparison to healthy males. This has been proven microscopically with a reduced diameter and deficient morphology of vascular components (Grant et al. 2013). The contraction of cavernosal smooth muscle cells is affected by hyperglycemia, with an increased forced response to vasoconstrictors. This could be partially explained because of a sensitization pathway in the protein Kinase C and the Rho A-Rho kinase Ca2+ pathways, which could cause a tendency towards a flaccid stage and modify the responses of nitric oxide (NO) (Chitaley et al. 2001). All of the above should be taken into account with the added factors that impact erectile function that include apoptosis or atrophy of the cavernous smooth muscle due to a diminished expression of bcl2, intracellular release of Ca2+, increased connective tissue proliferation due to tumor growth factor beta causing fibrosis and a deficient response to NO in the cavernous and sinusoidal artery with a decrease in the neuronal and endothelial levels of nitric oxide synthetase (Burchardt et al. 2000). In brief, there are several components that take place in the endothelial and neural damage in the periphery and central nervous system, which globally impact on erections in patients with DM.

To determine the degree of ED in DM patients there is the International Questioner for Erectile Function, that helps to determine the degree of ED, and evaluate the progression or response to the medical treatment. In certain cases, in which a more precise evaluation of the vascular flows is needed, an echo-Doppler could be used to determine the cavernous artery flux and morphology. Some research papers have proposed the use of nocturnal penile tumescence test and electro-stimulation studies to determine the damage of the myelinated sensory fibers of the pudendal somatosensory fibers in the lower extremity as well as unmyelinated fibers (Dean and Lue 2005).

It is very important to discuss the different treatment options with the patient and their partners in order to better understand their needs, worries and perspectives of their treatment. The first line of treatment is oral medications, followed by intracavernosal injection and finally with penile prosthesis surgery.

Sildenafil, tadalafil, udenafil and vardenafil are the four oral agents used for the ED treatment. They all share the same mechanism of action, which involves the hydrolysis of guanosine monophosphate to guanosine 5′ – monophosphate, diminishing it, then there is an increase in the relaxation of the cavernosal smooth muscle mediated by NO, increasing the blood flow in the corpus cavernosum resulting in penile erection. Boulton et al. demonstrated a significant improvement in the sexual function of men with type 2 DM (in the rigidity maintenance and satisfaction of the erection reported by the patient and their partner) (Boulton et al. 2001). Nevertheless, studies demonstrate that type 5 phosphodiesterase inhibitors are not as effective in men with ED without diabetes (Carson 2003).

The other difference in the several available drugs is the time and the length of the effect and side effects. It has recently been reported that the use of daily phosphodiesterase 5 inhibitors can improve not only the sexual function of patients but also diminishes their urinary tract symptoms associated with prostate enlargement, this could be explained by the existence of nitric oxide receptors in the bladder neck which impact on the relaxation resulting in a better micturition (Bittencourt et al. 2009). Vacuum erection devices and external support devices cause blood flow to be directed into the penis, and when a satisfactory erection is obtained, a compressive device is applied at the base of the penis in order to prevent a blood return and lose the erection. Among its limitations is that the penis is cold due to non-circulating blood, the sensation is diminished because nerve compression and the uncomfortable process to obtain the erection. There are few studies with the use of vacuum erection devices in diabetic patients, however, it has been reported by ED diabetic patients that they significantly improved their sexual intercourse with this device, however, only between 20 to 50% had satisfactory erections (Levine and Dimitriou 2001). The intraurethral suppositories are prostaglandin E1 suppositories that were injected into the urethra and distributed along it; however, its use has been limited because it is not worldwide distributed. It has a reported efficiency of 60% with diabetic men having a satisfactory sexual intercourse, although in clinical practice it has not been as effective (Huang and Lie 2013; Fulgham et al. 1998).

Prostaglandin E-1 injection directly into the corpus cavernosum has a direct effect on the blood vessels causing an immediate penile erection, with a reported response rate of as high as 83%, with a significantly improve in their sexual function (Heaton et al. 2001). Among its limitations, are the injection prior to each sexual encounter, this impact on the spontaneity of the relation, and its adverse effect of penile pain, hematomas, infection, fibrosis and priapism. Nevertheless, there are reports that there is a high compliance with patients injecting themselves for up to 7 continuous years despite the pain associated with its administration (16 of 18 ED diabetic males compared to 7 out of 22 healthy ED patients) (Perimenis et al. 2001; Basu and Ryder 2004).

### Urinary tract infections

It is well recognized that diabetic patients have a higher incidence of infections, and the urinary tract (UTI) is not the exception, with a large variety of infections. DM is associated with severe cutaneous infections of the genitals such as Fournier’s gangrene, and DM type 1 patients tend to have a higher incidence of pyelonephritis.

Bacterial cystitis is frequently suffered by diabetic patients; it is more common in women than in men, especially in those with type II DM. Diabetic women have a higher prevalence of asymptomatic bacteriuria than healthy women, and they have a greater tendency for developing symptomatic UTI and recurrent complications with a higher incidence of more serious complications (Geerlings et al. 2000a,[b]). Because of the above there is a tendency towards close screening and treatment of asymptomatic bacteriuria, however it has not been proven to diminish the number of symptomatic infections or hospitalizations (Harding et al. 2002).

Hospitalization due to pyelonephritis occurs more frequently in diabetic patients and they are at a higher risk of developing acute pyelonephritis (sometimes with bilateral renal involvement), which could progress to renal abscess, pyelitis or emphysematous cystitis or pyelonephritis and bacteremia. Pyelonephritis in DM can be the origin of severe complications that can end up in organ failure and even death (Stapleton 2002). Because of the above it is important to be vigilant of the usual clinical manifestations which include urinary urgency, frequency, bad odor, pain, tenesm, incomplete emptying and incontinence.

Despite being *E. coli* the most frequent bacteria, unusual and aggressive pathogens are more prevalent in DM such as: fungal infections, *Klebsiella*, gram negative rods, enterococci, group B streptococci, *Pseudomonas* and *Proteus mirabilis* (Ronald 2002). Premenopausal and postmenopausal periods double the risk of developing the UTI. Another risk factor is sexual activity, which is the most important risk factor in women with type I diabetes (Geerlings et al. 2000a,[b]). Strategies to prevent recurrent UTI are: postcoital antibiotics or prophylactic antimicrobials taken on a regular basis at bedtime, being considered trimethoprim, co-trimoxazole, or nitrofurantoin as the standard regimens (Grabe et al. 2009). Finally, it has been observed that diabetic women have up to four times the risk when they are in oral treatment or insulin (Boyko et al. 2002).

DM also results in abnormalities in the host defense system that may result in a higher risk of developing infection. Immunologic impairments such as defective migration and phagocytes alterations of chemotaxis in polymorphonuclear leukocytes are well common in diabetic patients. However, there is a study of diabetic women that found no differences in polymorphonuclear leukocyte function among women with diabetes, compared to healthy individuals (Dalal et al. 2009). Additionally certain cytokines such as IL-6 and other proinflammatory cytokines are diminished in the urine in comparison to women with no diabetes (Caqueiro et al. 2012).

## Conclusion

There is worldwide an epidemic of obesity, which increases significantly the number of diabetic patients, and if we consider that urologic diseases are very prevalent in the general population, it is very important to know which are the most frequent association of both, in order to diagnose them early and keep them in mind when evaluating these patients.

Even though incontinence and sexual dysfunction does not cause any life threatening disease, however it does cause severe discomfort and affects their quality of life. Because of this it is important to evaluate the sexual sphere when diabetic patients are treated for any other disease. The pathophysiology and possible mechanisms by which DM patients have more urologic complications are not clear, the genitourinary organs have a multicellular make it unlikely that a single mechanism underlies both voiding and sexual dysfunction in diabetes. The hyperglycemia impact on several levels of nerve, epithelium and mesenchymal components that is why is important to develop multiple therapeutic classes that may be relevant during specific phases of diabetes.

A better knowledge of the different influencing factors will provide a better opportunity to treat these patients. The common urologic treatments among diabetic patients should be carefully examined, as this is a disease that affects many organs at different levels and diabetes development is related to glycemic control of each individual, so it is important to consider the condition of each patient to select the most appropriate treatment.

It is important to keep in mind these complications in order to prevent them, diagnose them at early stages and treat them with an integral perspective of the individual, in order to offer the patients with DM a better quality of life.
